# Editorial: Social Cognition, Motivation, and Interaction: How Do People Respond to Threats in Social Interactions?

**DOI:** 10.3389/fpsyg.2017.01577

**Published:** 2017-09-27

**Authors:** Eva Jonas, Christina Mühlberger

**Affiliations:** Division of Social Psychology, Department of Psychology, University of Salzburg, Salzburg, Austria

**Keywords:** threat, discrepancy, motivational-affective state, motivated cognition, motivated behavior, Loop2Loop model

When people are in social interactions they often have to cope with different kinds of threats. Threats can result from interaction partners' concrete behaviors, like if they threaten our freedom to act or degrade our behavior. Further, threats can also be abstract or existential in nature, like when we sense a loss of control or meaning in a puzzling situation. These various forms of threats play important roles in our daily interactions and often change how we think and behave around others. In this special issue, we collected research approaching the topic of threat in social interactions from different perspectives. Now we step back and reflect on how these perspectives might be integrated into one broader framework.

## Sources of threat

Threat in social interactions can result from different sources: It can result from the *behavior of our interaction partners* and as such strongly influence our emotions, cognitions, and behavior, as when one feels ignored or excluded by the interaction partner (Wesselmann et al.), if the partner threatens one's freedom to act (Sittenthaler et al.; Steindl and Jonas; Niesta-Kayser et al.), uses unpleasant communication (Klonek et al.; Traut-Mattausch et al.), or appears to be deceptive or untrustworthy (Mackinger et al.).

However, threats can also result from *situational aspects* that are largely unrelated to the behavior of the interaction partner, like when the situation is socially uncertain (Gollwitzer et al.), puzzling (van den Bos et al.), involves vulnerability and the willingness to accept risks (Keller et al.) or poses a social-evaluative threat to the self (Frisch et al.). Moreover, one feels the pressure to improve one's performance (Scholl et al.) or one is in danger to lose a powerful position to the interaction partner (Scheepers et al.).

People can also enter an interaction situation by *having previously been affected by a threat* resulting from an unrelated event, in which they lost control (Stollberg et al.), had been reminded of their own mortality (Agroskin et al.), were exposed to goal conflicts (McGregor et al.), or feared to not fulfill one's duties and obligations (Keller et al.).

Finally, people can also feel threatened because of certain *predispositions*, like being in a prevention focus (Keller et al.; Scholl et al.), having high victim sensitivity (Gollwitzer et al.), or an anxious attachment style (Ein-Dor).

Despite the variation in circumstances, threatening situations always involve the experience of a discrepancy (Jonas et al., 2014), which means a discrepancy between the reality one is confronted with and what one would have otherwise wanted or expected. Broadly speaking one could sort these discrepancies as follows.

## Threats as the experience of discrepancies

### Discrepancy between motive and reality (perception of the actual situation)

Employees want to fulfill their job duties well but they are confronted with customer complaints (Traut-Mattausch et al.). Bank customers search for advice and would like to trust their financial consultant (Mackinger et al.). People wish to stick to their habits but are confronted with a change request (Klonek et al.). They strive for power but experience that they lack stable power (Scheepers et al.). People generally want to reach their goals, but the actual situation carries with it the prospect of failure (Frisch et al.; Keller et al.; Scholl et al.).

### Discrepancy between cognitive focus and reality (perception of the actual situation)

People expect to possess a certain freedom but experience that this freedom is threatened (Steindl and Jonas; Niesta-Kayser et al.; Sittenthaler et al.). People expect to be included into a group but are excluded (Wesselmann et al.).

### Discrepancy between motive and cognitive focus

Victim sensitive persons desire to trust but expect untrustworthinesss (Gollwitzer et al.) and insecurely attached people desire safety but expect threats (Ein-Dor). People strive for self-preservation, control, certainty, or meaning but have been reminded that they are mortal (Agroskin et al.), sometimes lack influence (Stollberg et al.), experience frustration and uncertainty (McGregor et al.), or experience confusion (van den Bos et al.).

In all these different forms, threats can be part of our daily interactions—and shape these interactions. When people are confronted with a threat it can have cascading consequences for their feelings, cognitions, and behaviors within a social interaction. In the different articles in this special issue, different sequences of central psychological variables (motivation, cognition, behavior depending on characteristics of the interaction situation, the behavior of the interaction partner, the motives and/or personalities of the acting person him-/herself) have been explored. We suggest connecting these different variables in a model of social interaction, which helps us to better understand the dynamic nature of social interactions (see Figure here).

## Threats in social interactions—the Loop2Loop model as dynamic perspective

According to Interdependence Theory (Kelley and Thibaut, 1978; Kelley et al., 2003), people transform the *actual* (objective) situation into an *effective* situation of idiosyncratic interpretations and meanings (see Figure 1 in Steindl and Jonas). To better understand this transformation process we suggest specifying people's motives, their cognitive focus in a specific situation, and their perception of the situation (see above and Figure here). The different papers in the special issue deal with a wide array of people's motives and needs, like their desire for control, certainty, meaning, belonging, freedom, and self-protection. The cognitive focus might be activated by situational aspects (e.g., a focus on gains vs. losses, or a focus on certain norms) or by personality characteristics. This focus can interact with people's perception of the actual situation and/or their motives as well as the motives can interact with the situation. The Loop2Loop Model of social interactions (see Figure here; see also Steindl and Jonas and Jonas and Bierhoff, 2017) suggests that as a result, the actual situation is transformed into an effective situation, i.e., how people subjectively construe a situation, which induces a certain motivational-affective state that is accompanied by motivated cognitions and often transforms into behavior. In a social interaction this behavior is visible to the interaction partner. Whether interaction partners feel threatened or valued within the interaction has important implications for how the interaction will unfold further, and these processes are explored next.

### Loop of motivational-affective states, motivated cognition, and motivated behavior

The experience of discrepancies result in a *motivational-affective state*, that can be related to the activation of two main motivational systems: the *behavioral inhibition system* (BIS, Gray and McNaughton, 2000), which is characterized by inhibition, anxious arousal, and attentional vigilance, and the (inversely related) *behavioral approach system* (BAS, Gray and McNaughton, 2000) characterized by an “impulse to go toward” (Harmon-Jones et al., 2013, p. 291). As described by McGregor et al., goal regulation can be described as interplay between the BIS and the BAS systems (see also Jonas et al., 2014). When the BAS is predominant people are in a process of unhindered goal-striving. They feel energized, free from anxious worries, concentrated and committed toward approaching their goal. When they are confronted with a discrepancy, BIS activity increases, ongoing goal-striving is inhibited, and people may feel greater anxiety. People want to leave this aversive state via motivational re-orientation. The activation of the BAS provides this orientation and helps to mute previous anxiety.

In support of this suggested process, Agroskin et al. demonstrates that participants high on trait need for closure reacted to mortality salience with increased BIS activity (measured i.a. by EEG), which in turn led to increased ethnocentrism and reluctance for cultural exploration. However, Scholl et al. and Scheepers et al. show that people can perceive a discrepancy as threat or challenge. When people think they lack sufficient personal resources (e.g., skills) to cope with the demands of the situation they experience threat because they evaluate their resources to be too low to overcome the discrepancy. In contrast, when people think they possess sufficient resources they experience challenge. This cognitive focus interacts with the features of the situation (e.g., stability of one's power) and together affects people's physiological responses and behavioral reactions to discrepancies. By making use of the Asch paradigm, van den Bos et al. demonstrate that situational circumstances can be so strong, for example when a certain experimental situation is very puzzling, that people can get stuck in the activation of the BIS. Similarly, Gollwitzer et al. argue that victim sensitive participants can get caught by anxiety, torn between anxiously showing attentional vigilance to threat related stimuli, and trying to avoid the threat. This can result in a motivational state of avoidance motivation. With regard to freedom threats, Niesta-Kayser et al. show that the activation of an avoidance goal can increase the experience of threat and in turn impair performance.

If people's goals and needs shape their thinking we call this *motivated cognition*. Motivated cognition can affect people's *perception* of the social situation, lead to biased *attention* to specific information, and guide *decision-making* (Hughes and Zaki, 2015). People can be engaged in motivated cognition in order to *serve an avoidance goal*. Such an avoidance orientation can lead to a defensive stance and help to explain, e.g., non-cooperative behavior. For example, victim-sensitive people who fear being exploited are more likely to interpret ambiguous cues as negatively foreboding in socially uncertain situations. This interpretive bias leads to mistrust which in turn increases uncooperative behavior (Gollwitzer et al.). In a similar vein, a prevention focus, characterized by feelings of worry, cautiousness, and wanting to prevent losses and failure (Keller et al.), is related to worry that others are dishonest or unreliable and to *motivated behavior*, such as transferring lower sums of money in a trust game. Similarly, Traut-Mattausch et al. found that when customer service representatives wanting to avoid talking to customers, they subsequently devalued the customer and his/her concerns/issues (motivated cognition). This devaluing by the representative increased degrading service reactions to the customer (motivated behavior).

As these aggressive responses could suggest, people might not only be motivated to further avoid the threatening stimulus but may also try to overcome the anxious state and activate the BAS and regain agency, which can be achieved by showing angry or aggressive responses directed toward the source of threat (Carver and Harmon-Jones, 2009) or by approaching rewarding stimuli. Jonas et al. (2014) proposed that if people think they are capable of changing the unpleasant situation (i.e., reduce the discrepancy to the desired end-state) they might attempt to resolve the threat through fairly direct efforts. Interestingly, in threat-related situations we often find an increase in *both*, anti- and pro-social responses. This is illustrated by Wesselmann et al. in the context of social exclusion. These authors suggest that in response to a threat a pro-social behavior is more likely if an inclusionary need (belonging and self-esteem) has been violated, and an anti-social response is more likely if a power/provocation need (control and meaningful existence) has been threatened. This threat-response mapping suggests that responses are aimed at restoration of a threatened need. Supporting this assumption, Stollberg et al. showed that loss of control threats increased identification with highly agentic groups, i.e., groups that served the threatened need, but not with low agentic groups. Furthermore, task-group identification mediated the increase in the feeling of collective control after a loss of personal control. Scholl et al. showed that depending on people's motivational-affective state, different interaction partners (high vs. low power groups) are preferred. For example, people often prefer interacting with those partners who fit one's regulatory focus, but threat leads people to prefer high power partners and thus may shift people's reliance away from dispositional preferences toward state deficits.

Yet, if a direct response to the threat is not available, people may respond in ways that do not resolve the threat but help to overcome the discrepancy in an indirect way. They may orient to a domain of pursuit that is separate from the source of discrepancy but that allows for renewed approach motivation and a restoration of a sense of agency. For example, in response to threat, people often increase their commitment to goals that seem unrelated to the threat. These goals can be categorized along the continua of personal to social and concrete to abstract in nature (see Jonas et al., 2014). Following existential threat people often affirm more abstract (personal or social) values, worldviews or group identifications—as illustrated by Agroskin et al. for increased ethnocentrism following mortality salience. Interestingly, McGregor et al. suggests that aggressive religious radicalization is often facilitated by concrete social defenses such as group aggression. Regardless of the domain, affirming the self, one's group and related goals can (at least partly) reduce the aversive feelings aroused by discrepancies.

To summarize, people's *experience of threat* depends on (a) their personality traits (e.g., some people are more sensitive toward experiencing discrepancies) and their motive strength, (b) situational circumstances and how they are perceived (e.g., situational uncertainties), and (c) what people focus on in a certain situation. People's *reactions to threat* seem to depend on similar variables, as McGregor et al. emphasize, like (a) their personality traits (e.g., some people are more prone toward experiencing anxiety and/or to show certain agentic responses, like for example aggression; furthermore people have different access to activate their internal resources to cope with a threat), (b) situational circumstances, and (c) what people perceive in a certain situation [affordances—what might be similar to people's cognitive focus (see Figure here)].

### Loop2Loop—consequences for social interactions

People's responses to threat influence how the social interaction further develops. When an actor exhibits aggressive behavioral intentions and evaluations following a freedom threat, this is related to the arousal of approach motivation (Sittenthaler et al.), which will have downstream impacts on their cognition and behavior toward the interaction partner moving forward. However, the actor's behavior will also influence the partner's motivational-affective state and his/her motivated cognition and behavior (e.g., an aggressively phrased complaint leads to an increase in people's heart rate, induces closed-mindedness, and an ignorance of improvement suggestions included in the complaint, Traut-Mattausch et al.). People's responses to threat can thereby contribute to a conflict loop and downward spiral. As another example of this process, Frisch et al. review literature that suggests that social-evaluative threats trigger stress reactions, such as neurophysiological responses and negative self-related cognitions. These stress reactions can have dysfunctional effects on social memory retrieval and socially adaptive behavior. If chronic stress prevents people from developing or employing the ability to adjust their behavior to other's needs, then this provides a source of interaction dissatisfaction. This could in turn induce mistrust, devaluation, and uncooperative behavior from the partner, which in turn might increase the threat the actor feels. In this way one loop leads to the next and in the end a negative interaction overall results, which is characterized by long-term biased cognition and interpersonal conflict. Indeed Steindl and Jonas found mistrust to be a mediator for uncooperative behavior following threat.

An interaction loop can evolve in a positive way if trustful behavior such as honesty or reliability creates a trustworthy atmosphere (Mackinger et al.), which in turn arouses a feeling of good prospects for one's motives. Klonek et al. found that reflective listening by a change agent (using Motivational Interviewing) helped to prevent the perception of threat with regard to changing one's environmental behavior. The more the agent's behavior was perceived to be empathetic, the more they came to believe that changing their behaviors might be beneficial.

Finally, Ein-Dor outlines to think about threat reaction processes at the group level with regard to personality styles. Specifically, he suggests that people with different attachment styles react to threats in different ways, and that the distribution of different styles within the group may help the group work together effectively to overcome threats. Some people are more sensitive to threat-related cues and show higher accuracy in detecting threats. Others are better in understanding how to deal with threats, like employing self-protective actions. Others yet are better able to coordinate group actions and motivate people to overcome the threat. Groups are effective if people who detect threats early alarm group members who are able to activate rapid responses. In this way social interaction between varied personality styles may help people to coordinate their activities and to discover ways to effectively behave together toward approaching common goals. The piece ultimately outlines some fascinating ways to consider how threats can be regulated effectively via social interaction for the benefit of each interaction partner.

## Conclusion

In this Research Topic, we presented research and theory connecting three levels of analysis (the social, the cognitive, and the motivational) and illustrated their functional interconnection in social interaction. The Loop2Loop model helps us to connect basic motivational processes, which manifest in neural processes related to behavioral inhibition vs. activation in a social situation, with social cognition, which shapes our attention and commitments toward belief systems, and people's behavior in social interactions, which can serve to motivate the reactions we receive from our interaction partners.

## Author contributions

EJ wrote the manuscript. CM contributed to and edited the manuscript. Both authors approved the final version of the manuscript for submission.

### Conflict of interest statement

The authors declare that the research was conducted in the absence of any commercial or financial relationships that could be construed as a potential conflict of interest.
